# Skeletal Muscle Gene Expression in Long-Term Endurance and Resistance Trained Elderly

**DOI:** 10.3390/ijms21113988

**Published:** 2020-06-02

**Authors:** Alessandra Bolotta, Giuseppe Filardo, Provvidenza Maria Abruzzo, Annalisa Astolfi, Paola De Sanctis, Alessandro Di Martino, Christian Hofer, Valentina Indio, Helmut Kern, Stefan Löfler, Maurilio Marcacci, Sandra Zampieri, Marina Marini, Cinzia Zucchini

**Affiliations:** 1 Department of Experimental, Diagnostic and Specialty Medicine, University of Bologna School of Medicine, 40138 Bologna, Italy; alessandra.bolotta3@unibo.it (A.B.); paola.desanctis@unibo.it (P.D.S.); marina.marini@unibo.it (M.M.); cinzia.zucchini@unibo.it (C.Z.); 2 IRCCS Fondazione Don Carlo Gnocchi, 20148 Milan, Italy; 3 Applied and Translational Research Center, IRCCS Istituto Ortopedico Rizzoli, 40136 Bologna, Italy; g.filardo@biomec.ior.it; 4 Giorgio Prodi Interdepartimental Center for Cancer Research, S.Orsola-Malpighi Hospital, 40138 Bologna, Italy; annalisa.astolfi@unibo.it (A.A.); valentina.indio2@unibo.it (V.I.); 5 Department of Morphology, Surgery and Experimental Medicine, University of Ferrara, 44121 Ferrara, Italy; 6 Second Orthopaedic and Traumatologic Clinic, IRCCS Istituto Ortopedico Rizzoli, 40136 Bologna, Italy; aledimartino75@gmail.com; 7 Ludwig Boltzmann Institute for Rehabilitation Research, 1160 Wien, Austria; christian.hofer@rehabilitationresearch.eu (C.H.); helmut@kern-reha.at (H.K.); stefan.loefler@rehabilitationresearch.eu (S.L.); 8 Department of Biomedical Sciences, Knee Joint Reconstruction Center, 3rd Orthopaedic Division, Humanitas Clinical Institute, Humanitas University, 20089 Milan, Italy; maurilio.marcacci@humanitas.it; 9 Department of Surgery, Oncology and Gastroenterology, University of Padua, 35122 Padua, Italy; sanzamp@unipd.it; 10 Department of Biomedical Sciences, University of Padua, 35131 Padua, Italy

**Keywords:** skeletal muscle, sarcopenia, aging, exercise, endurance and resistance training, gene expression

## Abstract

Physical exercise is deemed the most efficient way of counteracting the age-related decline of skeletal muscle. Here we report a transcriptional study by next-generation sequencing of vastus lateralis biopsies from elderly with a life-long high-level training practice (*n* = 9) and from age-matched sedentary subjects (*n* = 5). Unsupervised mixture distribution analysis was able to correctly categorize trained and untrained subjects, whereas it failed to discriminate between individuals who underwent a prevalent endurance (*n* = 5) or a prevalent resistance (*n* = 4) training, thus showing that the training mode was not relevant for sarcopenia prevention. KEGG analysis of transcripts showed that physical exercise affected a high number of metabolic and signaling pathways, in particular those related to energy handling and mitochondrial biogenesis, where AMPK and AKT-mTOR signaling pathways are both active and balance each other, concurring to the establishment of an insulin-sensitive phenotype and to the maintenance of a functional muscle mass. Other pathways affected by exercise training increased the efficiency of the proteostatic mechanisms, consolidated the cytoskeletal organization, lowered the inflammation level, and contrasted cellular senescence. This study on extraordinary individuals who trained at high level for at least thirty years suggests that aging processes and exercise training travel the same paths in the opposite direction.

## 1. Introduction

Sarcopenia—the major cause of frailty and falls in elderly—is characterized by a decline in mass, strength, and function of skeletal muscle. Several combined factors underlie the establishment of sarcopenia, including a decline in muscle stem cells [1,2,3,4,5], the occurrence of mitochondrial dysfunction [6,7], hormonal deregulation [8] and the activation of inflammatory signal pathways [9,10].

It is known that the amount of physical performance declines with age and at the same rate in male and female [11,12]. While lower levels of exercise are at the same time both a consequence and a relevant contributing cause to sarcopenia, it is well established that physical exercise is an effective countermeasure to age-related muscle wasting [13,14]. However, although resistance training (RT) might achieve superior results in preserving the muscle mass [15], the uncertainties regarding the molecular pathways involved in the establishment of sarcopenia lead to uncertainties on which exercise is more suitable to counteract muscle aging [16]. A large body of literature examined the effects of different modalities of exercise in a wide variety of health conditions; recently, Pillon et al. [17] performed a meta-analysis where over 60 transcriptomic studies, carried out with different training regimens, in different skeletal muscles and different human populations, were examined by the aid of a sophisticated software; in this way they identified the more representative genes whose expression was associated with what they named “the skeletal muscle response to exercise and inactivity”.

We took a different approach, by taking advantage of the availability of skeletal muscle biopsies from an exceptional group of senior (65–79 years of age) amateur athletes with a life-long practice of high-level physical activity [18]. We report here the results of a transcriptional study using Next Generation Sequencing (NGS) performed on vastus lateralis (VL) bioptic specimens obtained from the above-mentioned senior athletes and from sedentary elder subjects (70–75 years of age), with the aim of identifying genes and related pathways affecting skeletal muscle preservation from the age-related decay. Moreover, we expected that the transcriptional study would help to understand the pathways by which one training regimen (endurance vs. resistance) might be more appropriate in counteracting age-associated sarcopenia.

## 2. Results and Discussion

### 2.1. Effects of Life-Long Training on General Fitness

The anthropometric values of the control sedentary subjects are summarized in Table 1 and are fully comparable with those of trained subjects, shown in Table 2 and Table 3. Nevertheless, the high training level of senior athletes (Table 2) led to very good performances in functional force tests (Table 3). 

Since control sedentary subjects (SED) were very limited in their movements due to knee problems, sedentary and trained subjects that were involved in this study represent, in same way, the two ends of a physical activity scale in their age class. Worth noting, the trained seniors (TRA) were a subset from a larger group of trained subjects described in other manuscripts authored by some of us [18,19].

Table 4 reports the mean fiber diameter of TRA subjects. Resistance-trained (RT) subjects showed significantly larger muscle fibers, in particular of the fast type, in comparison to endurance-trained (ET) subjects (*p* values are in bold when significantly different between RT and ET subjects). No difference in slow type fiber diameter was observed between the two groups of trained subjects.

### 2.2. Differentially Expressed Genes

The gene expression profile of TRA was compared with that of SED subjects (Table S1, sheet A). Out of 13,589 expressed genes, 7423 were differentially expressed at *p* ≤ 0.01. Of them, 3889 were downregulated and 3534 upregulated in TRA versus SED VL. Noteworthy, most of them (6644) had an adjusted *p* value ≤ 0.01, while for the remaining 779 the adjusted *p* value was slightly higher (0.01 ≤ adj *p* value ≤ 0.02). Since the statistical analyses are more reliable when performed with a larger set of data, all analyses were performed on the whole set of 7423 genes. The comparison between ET and RT subjects yielded a list of 331 out of 12,251 differentially expressed genes with statistical significance *p* ≤ 0.01, but none had an adjusted *p* value ≤ 0.05 (Table S1, sheet B). No overlap was found between the list of the most up- and down-regulated genes and that published by Pillon et al. [17].

### 2.3. Mixture Distribution Analysis and Differences between ET and RT Training

**Figure 1:**
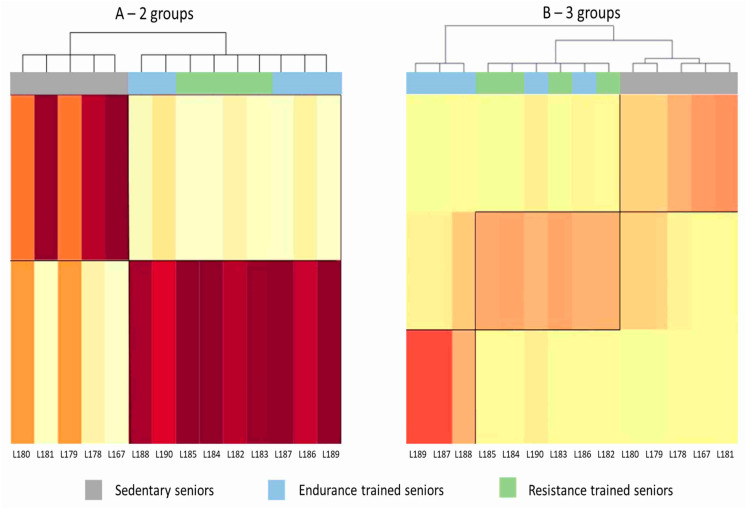
Heat maps generated by mixture distribution analysis. Each column represents one subject. The darker the color shade, the closer is the affinity of the subject’s pattern of gene expression to that characterizing the group to which he was assigned by the cluster analysis. (**A**) Unsupervised analysis clustered subjects in two groups, SED and TRA, without being able to discriminate between ET and RT athletes. (**B**) Trying to group the subjects in three clusters (SED, ET and RT), mixture analysis misclassified two ET subjects, who were assigned to the RT group.

Unsupervised mixture distribution analysis clustered SED and TRA subjects, without being able to discriminate between RT and ET athletes (Figure 1A), thus showing that the training activity produces a significant change in the expression profile of the trained subjects compared to the sedentary subjects, regardless of the type of training. By specifying the presence of three groups (SED, RT and ET), mixture analysis misclassified two ET subjects (L186 and L190), thus suggesting that the two modes of training influence the gene expression profile in a largely overlapping way (Figure 1B).

The R statistics tool assigned the difference between RT and ET subjects to only 20 characterizing genes (Table 5); all of them were more expressed in the correctly assigned ET subjects than in the RT subjects. Of note, none of these genes appears to be functionally related to the specific characteristics of the training; nevertheless, it may be interesting to note that at least half of them code for proteins involved in nuclear regulation. Curiously, five genes, namely NR4A3, ANKRD1, ATF3, FOS, Cyr61, were included in the set identified by Pillon et al. [17] as characterizing acute exercise, both endurance and resistance, whereas a sixth gene-GADD45a-involved in DNA repair and upregulated by environmental stressors, was in Pillon et al. analysis identified as characteristic of inactivity. It is possible that this discrepancy depends on whether the age group 70–79 years is not sufficiently represented within the data analyzed in [17], or whether, as pointed out earlier, subjects studied here represent the two ends of a physical activity scale in their age class. Nine genes (Table 6) prevented the correct clustering of two ET subjects-L186 and L190, but only six out of them were part of the characterizing subset of 20 genes mentioned above. The missed assignment of subjects L186 and L190 to the ET group was not apparently based on anthropometric or training features. Rather, since at least three out of the nine genes responsible for the incorrect clustering of two out of five ET subjects—HBEGF, IER5 and MAOA—are controlled by circadian rhythms, it is not unlikely that having missed to plan the biopsies in a predefined time might have hindered a number of gene expression differences between the ET and the RT subjects, such as those listed in Table 5.

It is generally thought that RT results in an increase in muscle mass, whereas ET results in an increase in muscle oxidative metabolism. In fact, in young subjects [20], ten weeks of RT stimulated myofibrillar protein synthesis and phosphorylation of Akt-mTOR-p70S6K proteins, while ET stimulated only mitochondrial protein synthesis. Data like these led to the development of the concept of concurrent training, meaning that either exercise regimen seems to hinder the training effects of the other [21], a concept that has largely influenced the working patterns of professional coaches. The underlying idea is based on the concept that RT activates the PKB/AKT/mTOR pathway, leading to protein synthesis and muscle hypertrophy, while ET activates the AMPK pathway, leading to mitochondrial biogenesis, increased glucose transport and other features characterizing oxidative metabolism. While such concepts had become paradigmatic, some experimental data challenged the idea that the two pathways were independent one from the other. In fact, Melov et al. [22] reported that RT reversed both mitochondrial impairment and muscle weakness in aged individuals; on the other hand, Abruzzo et al. [23] showed that ET led to a moderate hypertrophy in slow-twitch muscles and to a shift towards a more oxidative phenotype in both slow- and fast-twitch muscles; similarly, Konopka and Harber [24] reported skeletal muscle hypertrophy following ET. Data reported here demonstrate that, at least in elderly, the long-time effect of high-level exercise training are, for many aspects, broadly indistinguishable on the basis of it being aerobic or anaerobic.

### 2.4. Differentially Expressed KEGG Profiles

The relevant functions linked to the differentially expressed genes in the comparison of TRA vs. SED were evaluated by KEGGs using Enrichr. Annotation analysis identified a number of significantly modulated pathways. The 15 highest scoring pathways (after excluding those related to pathologies and those not relevant to the cell model) are shown in Table 7, while the 25 highest scoring KEGGs, including those we estimated to be not relevant for our biological model, the list of differentially expressed genes and the supporting statistics are shown in Table S2. For each relevant KEGG pathway, Table S3 reports the gene expression data of differentially expressed genes, ordered according to the value of logFc.

For description and discussion purposes, we functionally grouped KEGG pathways, in order to outline cellular processes; in some instances, the analysis was extended to include biological processes and other pathways beyond the highest scoring ones. This also helped in drawing comparisons with two recent works reporting proteomics data from human VL, where 58 healthy persons aged 20 to 87 years and characterized by four different levels of physical activity were studied [25,26]. It was strikingly evident that most age-related changes in skeletal muscle protein expression described in ref. [26] were found to be reversed in our study, where the cognate mRNAs from aged athletes were examined and compared to those of aged-matched sedentary subjects, thus highlighting the exercise-operated reversal of aging processes, when present. Of note, this observation somehow lends greater legitimacy to the interpretation of transcriptomics data, provided that one keeps in mind that the half-life of proteins and messengers may be different.

#### 2.4.1. Energy Usage-Related Pathways

**Figure 2:**
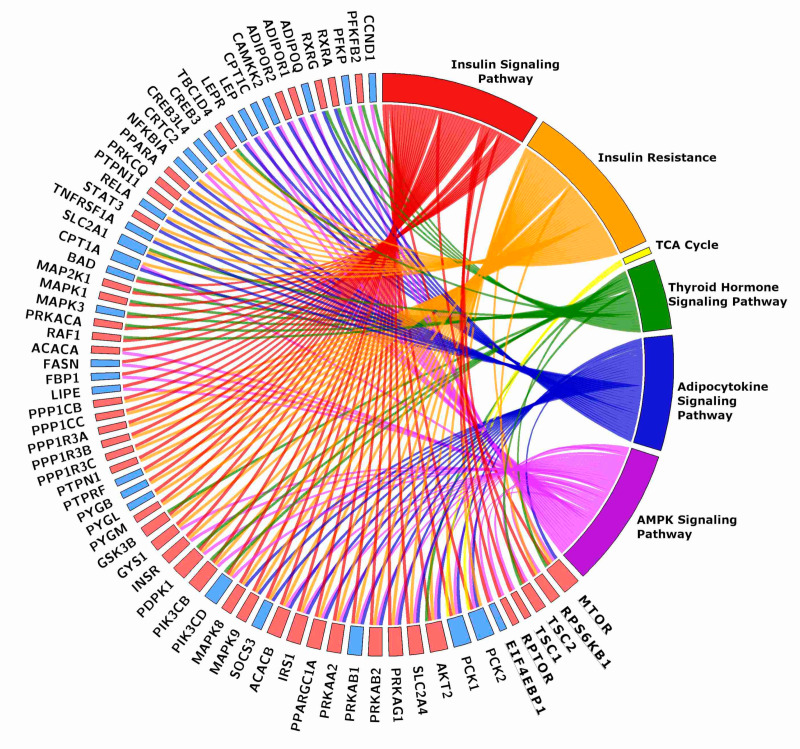
Chord plot showing the genes shared by two or more energy-related KEGGs. The rectangle flanking the gene name is red when the gene is more expressed in TRA than in SED VL, blue when it is less expressed. The plot includes six genes of the mTOR pathway, which were grouped together for clarity sake. Gene names and details on the pathways are in the text.

As expected, physical activity greatly affected energy usage pathways, promoting ATP production against gluconeogenesis. Metabolic pathways are intertwined, share a large number of genes, interact with many different processes, and affect the activity and the number of mitochondria, the rate of protein synthesis, the level of autophagy, among others. Figure 2 shows the intertwining of energy usage-related pathways and the genes they collectively affect. Results and discussion will be grouped in paragraphs for greater clarity.

#### Insulin Signaling Pathway and Insulin Resistance 

**Figure 3:**
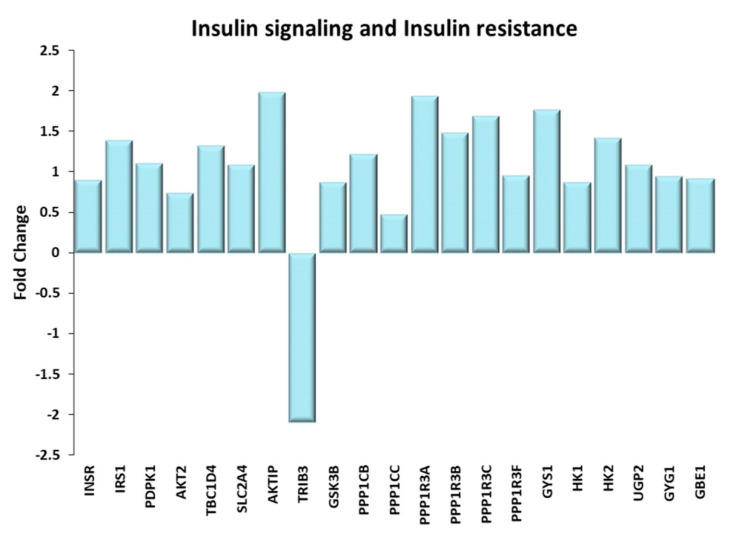
Insulin signaling and Insulin resistance. Genes discussed in the text are shown here as fold change in the comparison of TRA and SED subjects. INSR: Insulin receptor; IRS1: Insulin receptor substrate 1; PDPK1: 3-phosphoinositide dependent protein kinase 1; AKT2: AKT serine/threonine kinase 2; TBC1D4: Akt substrate of 160 kDa or AS160; SLC2A4: glucose transporter type 4 or GLUT-4; AKTIP: AKT interacting protein; TRIB3: tribbles pseudokinase 3; GSK3B: glycogen synthase kinase 3 beta; PPP1CB, PPP1CC: protein phosphatase 1 catalytic subunit beta, gamma; PPP1R3A, PPP1R3B, PPP1R3C, PPP1R3F: protein phosphatase 1 regulatory subunit 3A, 3B, 3C, 3F; GYS1: glycogen synthase 1; HK1, HK2: Hexokinases 1, 2; UGP2: UDP-glucose pyrophosphorylase 2; GYG1: glycogenin; GBE1: Glycogen branching enzyme.

The insulin signaling (Figure 3) pathway controls the glucose homeostasis by modulating the glucose uptake and regulating the glycogen synthesis, the glycolysis and the gluconeogenesis [27]. Both acute and chronic physical exercise stimulate the insulin-signaling pathway and the glucose uptake, independently of the training modality [28]. Gene expression data presented here are in keeping with the literature data.

In fact, all steps involved in Insulin signaling were found to be transcriptionally upregulated in TRA VL, starting with Insulin Receptor, Insulin Receptor Substrate 1, kinases PDPK1 and AKT serine/threonine kinase 2, continuing with a downstream target of AKT termed “Akt substrate of 160 kDa” involved in the expression and translocation of glucose transporter type 4 (GLUT4) to plasma membrane [29], and ending with the glucose transporter itself. The picture is completed by the upregulation of the AKT enhancer AKTIP [30] and by the downregulation of the AKT2 inhibitor TRIB3 [31], the key activator of the insulin resistance pathway in muscle cells.

A critical contribution to the upregulation of GLUT4 comes also from the Ca^2+^/Calmodulin signaling pathway [32]. As expected, exercise increased calcium metabolism in muscle cells, by upregulating plasma membrane and mitochondrial transporters, voltage-dependent anion-selective channels, and sarcoplasmic reticulum Ca^2+^ ATPase. Intracellular calcium binds to calmodulin (not transcriptionally upregulated in TRA VL), which in turn activates the Ca^2+^/calmodulin-dependent protein kinases. Downstream components of this pathway are members of the histone deacetylase family and of the myocyte enhancer factor 2 family (see paragraph “Calcium Involvement in Energy-Related Processes” below).

Glucose can be stored in the muscle in the form of glycogen or used to produce energy (ATP). The glycogen synthesis is finely regulated by the insulin-signaling pathway, mainly through phosphorylation and de-phosphorylation events [33]; nevertheless, we found that the activation of key enzymes was also played at the transcriptional level; in fact, glycogen synthase kinase 3 beta and the catalytic and regulatory subunits of protein phosphatase 1, which are downstream of PKB/AKT activity, as well as glycogen synthase, the rate-limiting enzyme in the glycogen synthesis, were all upregulated in TRA VL. In addition, the up-regulation of other genes involved in the glycogen synthesis, such as hexokinases, UDP-glucose pyrophosphorylase 2, glycogenin and the glycogen branching enzyme, corroborated the activation of the glycogen synthesis pathway.

#### Energy Production

**Figure 4:**
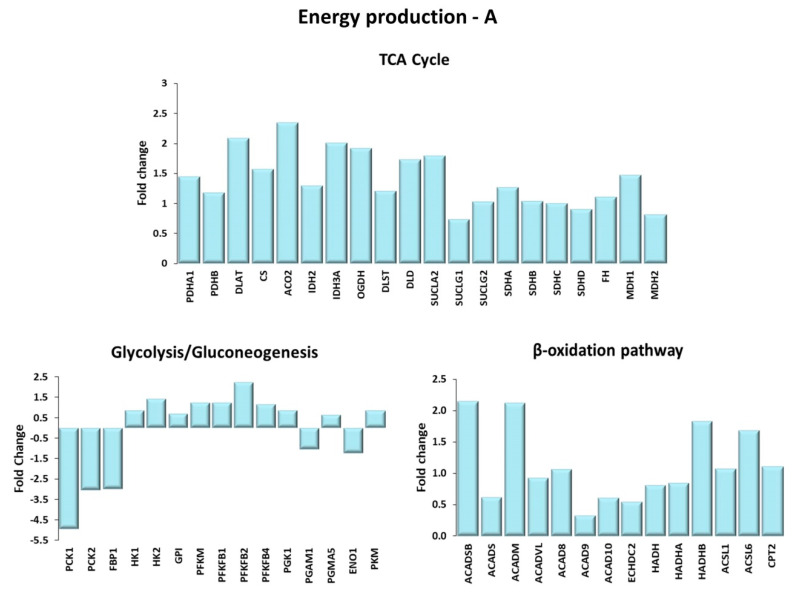

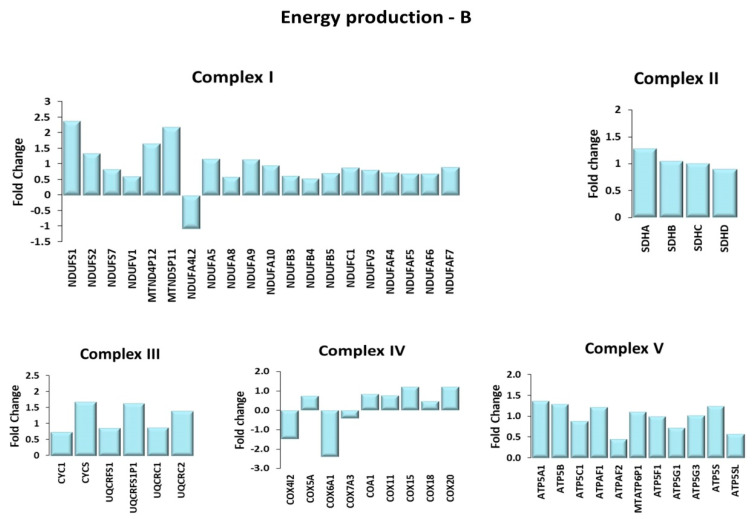
Energy production. Genes are shown here as fold change in the comparison of TRA and SED subjects. (**A**) TCA cycle. PDHA1, PDHB: pyruvate dehydrogenase E1 subunit α 1, β; DLAT: Dihydrolipoamide S-acetyltransferase; CS: citrate synthase; ACO2: aconitase 2; IDH2: isocitrate dehydrogenase (NADP+)2; IDH3A: isocitrate dehydrogenase (NADP+)3 catalytic subunit α; OGDH: oxoglutarate dehydrogenase; DLST: dihydrolipoamide S-succinyltransferase; DLD: dihydrolipoamide dehydrogenase; SUCLA2: succinate-CoA ligase ADP-forming subunit β; SUCLG1: succinate-CoA ligase GDP/ADP-forming subunit α; SUCLG2: succinate-CoA ligase GDP-forming subunit β; SDHA: succinate dehydrogenase complex flavoprotein subunit A; SDHB: succinate dehydrogenase complex iron sulfur subunit B; SDHC: succinate dehydrogenase complex subunit C; SDHD: succinate dehydrogenase complex subunit D; FH: fumarate hydratase; MDH1 and MDH2: malate dehydrogenase 1 and 2. Glycolysis/Gluconeogenesis. PCK1, PCK2: phosphoenolpyruvate carboxykinase 1, 2; FBP1: fructose-bisphosphatase 1; HK1, HK2: hexokinases 1, 2; GPI: glucose-6-phosphate isomerase; PFKM: phosphofructokinase muscle; PFKFB1, PFKFB2, PFKB4: 6-phospho-fructo-2-kinase/fructose-2,6-biphosphatase 1, 2, 4; PGK1: phosphoglycerate kinase 1; PGAM1, PGMA5: phosphoglycerate mutase 1, 5; ENO1: enolase 1; PKM: pyruvate kinase. β-oxidation pathway. ACADSB: acyl-CoA dehydrogenase short/branched chain; ACADS: acyl-CoA dehydrogenase short chain; ACADM: acyl-CoA dehydrogenase medium chain; ACADVL: acyl-CoA dehydrogenase very long chain; ACAD8, ACAD9, ACAD10: acyl-CoA dehydrogenase family member 8, 9, 10; ECHDC2: enoyl-CoA hydratase domain containing 2; HADH: hydroxyacyl-CoA dehydrogenase; HADHA, HADHB: 3-ketoacyl-CoA thiolase α, β; ACSL1, ACSL6: acyl-CoA synthetase long chain family member 1, 6; CPT2: carnitine palmitoyl transferase-2. (**B**) Mitochondrial electron transport chain. Complex I: NDUFS1, NDUFS2, NDUFS7, NDUFV1: NADH:ubiquinone oxidoreductase core subunit S1, S2, S7, V1; MTND4P12: mitochondrially encoded NADH dehydrogenase 4 psuedogene 12; MTND5P11: mitochondrially encoded NADH dehydrogenase 5 psuedogene 11; NDUFA4L2: NDUFA4 mitochondrial complex associated like 2; NDUFA5, NDUFA8, NDUFA9, NDUFA10, NDUFB3, NDUFB4, NDUFB5, NDUFC1, NDUFV3: NADH:ubiquinone oxidoreductase subunit A5, A8, A9, A10, B3, B4, B5, C1, V3; NDUFAF4, NDUFAF5, NDUFAF6, NDUFAF7: NADH:ubiquinone oxidoreductase complex assembly factor 4, 5, 6, 7. Complex II: SDHA: succinate dehydrogenase complex flavoprotein subunit A; SDHB: succinate dehydrogenase complex iron sulfur subunit B; SDHC: succinate dehydrogenase complex subunit C; SDHD: succinate dehydrogenase complex subunit D. Complex III: CYC1: cytochrome c1; CYCS: cytochrome c, somatic; UQCRFS1: ubiquinol-cytochrome c reductase, Rieske iron-sulfur polypeptide 1; UQCRFS1P1: ubiquinol-cytochrome c reductase, Rieske iron-sulfur polypeptide 1 pseudogene 1; UQCRC1 and UQCRC2: ubiquinol-cytochrome c reductase core protein 1 and 2. Complex IV: COX4I2, COX5A, COX6A1: cytochrome c oxidase subunit 4I2, 5A, 6A1; COX7A3: cytochrome c oxidase subunit 7A2 pseudogene 2; COA1: cytochrome c oxidase assembly factor 1 homolog; COX11: cytochrome c oxidase copper chaperone COX11; COX15: cytochrome c oxidase assembly homolog COX15; COX18, COX20 cytochrome c oxidase assembly factor COX18, COX20. Complex V: ATP5A1, ATP5B, ATP5C1: ATP synthase F1 subunit α, β, γ; ATPAF1, ATPAF2: ATP synthase mitochondrial F1 complex assembly factor 1, 2; MTATP6P1: MT-ATP6 pseudogene 1; ATP synthase peripheral stalk-membrane subunit b; ATP5G1, ATP5G3: ATP synthase membrane subunit c locus 1, locus 3; ATP5S: distal membrane arm assembly complex 2 like or DMAC2L; ATP5SL: distal membrane arm assembly complex 2 or DMAC2.

Differentially expressed genes involved in energy production are shown in Figure 4A,B. Besides being stored as glycogen, glucose can be used for ATP production through the respiratory pathway, which includes the glycolysis, the tricarboxylic acid (TCA) cycle and the mitochondrial electron transport chain. The insulin signaling pathway positively regulates the glycolytic enzyme gene expression [34]. With the exception of the genes encoding the enzymes phosphoenolpyruvate carboxykinase and fructose 1,6 biphosphatase, key enzymes involved in gluconeogenesis, the other genes of this pathway were up-regulated in TRA VL compared to SED VL, confirming that physical exercise enhanced ATP production through the activation of the respiratory pathway. In support of this, we found that genes encoding the enzymes involved in glycolysis, including hexokinases, 6-phosphofructo-2-kinase and pyruvate kinase, were up-regulated in the VL of TRA subjects. Moreover, the mRNA amount of the genes encoding the different subunits of the mitochondrial electron transport chain complexes was increased in the VL of TRA subjects.

The TCA pathway is fueled by the acetyl-CoA, which is generated not only by the conversion of pyruvate, obtained from glycolysis via the pyruvate dehydrogenase complex, but also by mitochondrial β-oxidation. In this study, the enzymes involved in the β-oxidation pathway, including acyl CoA dehydrogenase, enoyl-CoA hydratase, L-3-hydroxyacyl-CoA dehydrogenase, and 3-ketoacyl-CoA thiolase were found to be transcriptionally upregulated. Moreover, the gene expression of other enzymes, which act upstream and are essential for the β-oxidation process, was increased by physical training. They include the muscle isoforms of the acyl-CoA synthetase, which catalyze the coupling of fatty acids with coenzyme A and the enzymes involved in the transport of fatty acyl-CoAs across the mitochondrial outer and inner membranes, such as carnitine palmitoyl transferase-2.

#### AMPK Activation 

**Figure 5:**
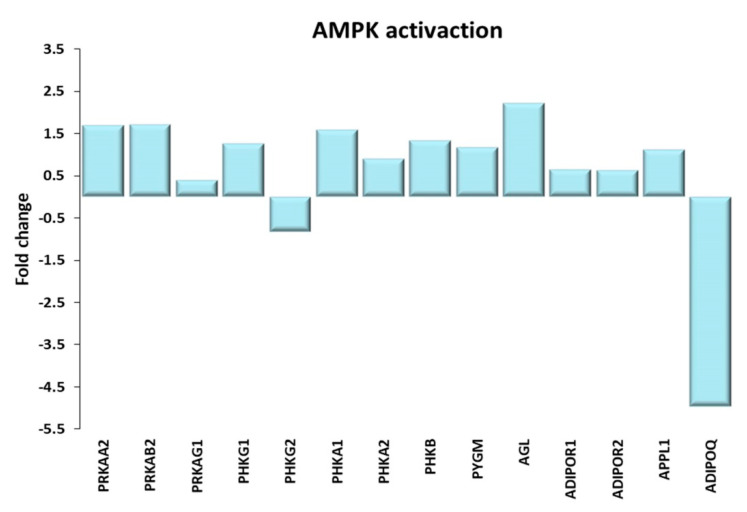
AMPK activation. Genes discussed in the text are shown here as fold change in the comparison of TRA and SED subjects. PRKAA2: Protein kinase AMP-activated catalytic subunit α 2; PRKAB2, PRKAG1: Protein kinase AMP-activated non-catalytic subunit β 2, γ 1; PHKG1, PHKG2: phosphorylase kinase catalytic subunit γ 1, 2; PHKA1, PHKA2, PHKB: phosphorylase kinase regulatory subunit α 1, α 2, β; PYGM, glycogen phosphorylase, muscle associated; AGL amylo-α-1, 6-glucosidase, 4-α-glucanotransferase; ADIPOR1, ADIPOR2: adiponectin receptor 1, 2; APPL1: adaptor protein, phosphotyrosine interacting with PH domain and leucine zipper 1; ADIPOQ: adiponectin.

Exercise leads to an increased energy expenditure, hence to an increase in the intracellular AMP/ATP ratio, which activates the AMP-activated protein kinase (AMPK), the central sensor of intracellular energy status [35]. The activation of AMPK (see Figure 5) depends on exercise intensity and duration [36,37]. AMPK is a heterotrimeric complex, containing a catalytic subunit (alpha), and two regulatory subunits (β and γ), each made up by different subunits [38], which are assembled to form different complexes depending on the type of exercise and duration [37]. In our model, we found an increase in the expression of the genes encoding the subunits α2, β2 and γ1, in a remarkable agreement with literature data [39].

Once activated, AMPK stimulates the muscle glucose uptake, increasing the expression and the translocation of GLUT4 to the plasma membrane [40] and stimulates glycogen [41]. We found an increase in the mRNA expression of both catalytic and regulatory subunits of the phosphorylase kinase and in glycogen phosphorylase mRNA in TRA VL. Moreover, the gene expression of other enzymes involved in the glycogen breakdown such as the glycogen debranching enzyme amylo-α-1,6-glucosidase, 4-α-glucanotransferase was found up-regulated in TRA VL.

AMPK activation not only depends on the AMP/ATP ratio, but also on other signaling pathways, including that mediated by adiponectin, a myokine produced by muscle tissue acting in a paracrine/autocrine manner through the binding to its receptors AdipoR-1 and AdipoR-2 [42,43,44]. Adiponectin regulates the metabolism through blood glucose control and fatty acid oxidation and indirectly through mitochondrial biogenesis, and has anti-inflammatory and antioxidant activities [45]. The adipocytokine pathway appears to be upregulated in TRA VL for what adiponectin is concerned. In fact, we found an increase in the expression of both AdipoR-1 and AdipoR-2 as well as of APPL1—their adaptor protein. On the contrary, the expression of adiponectin itself was down-regulated in TRA subjects; this result might be explained by the fact that adiponectin expression is more elevated in the fast-twitch oxidative type IIA and IID fibers than in slow-twitch oxidative fibers I [46], while, as reported in Zampieri et al. [19], the trained subjects enrolled in our study showed a significantly higher percentage of slow-type fibers compared to age-matched sedentary individuals.

#### Mechanistic Target of Rapamycin (mTOR) Pathway

**Figure 6:**
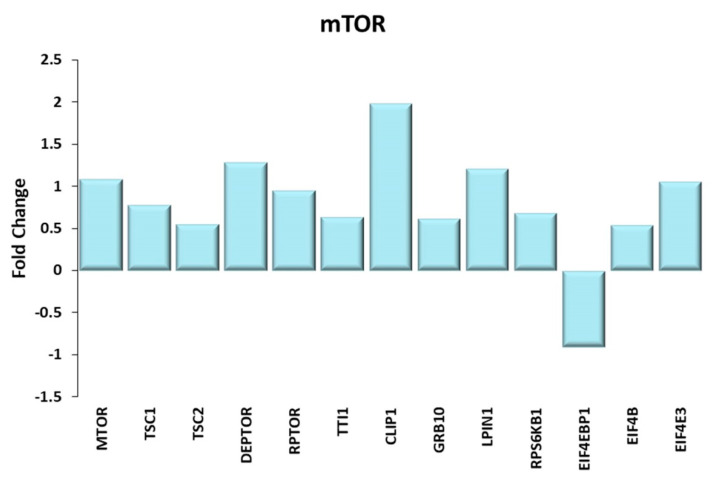
mTOR. Genes discussed in the text are shown here as fold change in the comparison of TRA and SED subjects. MTOR: mechanistic target of rapamycin kinase; TSC1 and TSC2: TSC complex subunit 1, 2; DEPTOR: DEP domain containing MTOR interacting protein; RPTOR: regulatory associated protein of MTOR complex 1; TTI1: TELO2 interacting protein 1; CLIP-1: CAP-Gly domain containing linker protein 1; GRB10: growth factor receptor bound protein 10; LPIN1: lipin 1; RPS6KB1: ribosomal protein S6 kinase B1; EIF4EBP1: eukaryotic translation initiation factor 4E binding protein 1 or 4E-BP1; EIF4B: eukaryotic translation initiation factor 4B; EIF4E3: eukaryotic translation initiation factor 4E family member 3.

Though not present within the 15 most representative KEGG, the mTOR pathway should be considered when discussing energy usage in TRA and in SED skeletal muscle. It is largely regulated by phosphorylation and dephosphorylation events; nevertheless, the emergence of a highly coordinated transcriptional regulation is striking, suggesting that it may be not coincidental. The mTORC1 pathway (Figure 6) is a hub to which a wide range of metabolic pathways converges; in fact, it is negatively regulated by the AMPK and positively regulated by the insulin pathway/AKT and by thyroid hormones (see Figure 2, where six genes of the mTOR pathway were grouped together) [47,48]. AMPK, transcriptionally upregulated and activated by energy expenditure, activates the TSC1/2 complex, which in turn inhibits the activation of mTORC1; a contribution to the inhibition of the mTORC1 complex is also due to DEPTOR [49]. Of note, TSC1, TSC2 and DEPTOR are all transcriptionally upregulated in TRA VL. Thus, on one side, the genetic upregulation of mTORC1 inhibitors may suggest the prevalence of AMPK-driven inhibition of the mTORC1 signal in TRA VL; on the other side, several genes of the mTORC1 complex (RAPTOR, mTOR, Tti1), as well as some of the genes positively controlled by mTORC1 (CLIP1, Grb-10, Lipin1, RPS6KB1) were upregulated in TRA muscles, presumably as a result of the activation of the insulin pathway. 

Among the processes laying downstream of mTOR, there is the pathway leading to increased CAP-dependent protein translocation, which depends on eIF4B and on eIF4Es, the former being activated by RPS6KB1-mediated phosphorylation and the latter being inhibited by 4E-BPs [50,51,52], which in turn is inhibited by mTORC1. While 4E-BP1 was found to be transcriptionally downregulated in TRA VL, both eIF4B and eIF4E3 were transcriptionally upregulated; noteworthy, eIF4Es are upregulated also downstream of the insulin signaling pathway, via IRS-1. The increase of protein synthesis mediated by insulin and mTOR signaling led to the increase in muscle mass observed in senior sportsmen by Zampieri et al. [19] and expected as a result of exercise training [24].

#### Ribosomes

Downstream the mTOR signaling, protein synthesis enhancement is fueled also by ribosome biogenesis. However, the Ribosome KEGG pathway is strikingly downregulated in TRA VL (see Table S3). Paradoxical downregulation of ribosomal gene RNAs was also described to occur in human exercised muscles as a function of their hypertrophy [53]. Of note, in the proteomic study quoted above [26], the downregulation of ribosomal proteins was identified as a hallmark of aging; when the same protein set was examined as a function of physical activity [25], no training-related change was observed in ribosomal protein amount. In contrast to this result, our data suggest that physical training further downregulated ribosomal gene expression in old subjects, in agreement with the results by Phillips et al. [53]. On the other hand, genes coding for the mitochondrial ribosomes were upregulated by training.

#### Mitochondria and NAD+/NADH Ratio

**Figure 7:**
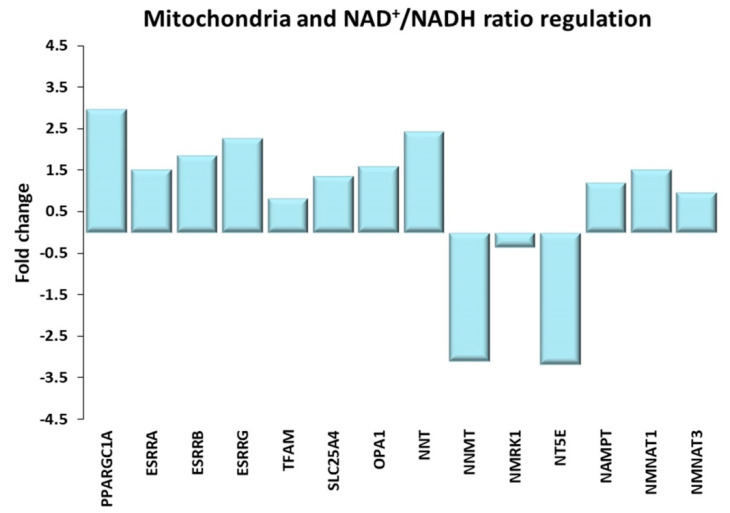
Mitochondria and NAD+/NADH ratio regulation. Genes discussed in the text are shown here as fold change in the comparison of TRA and SED subjects. PPARGC1A: proliferator-activated receptor γ coactivator-1 α or PGC1α; ESRRA, ESRRB, ESRRG: estrogen related receptors α, β, γ; TFAM: mitochondrial transcription factor A; SLC25A4: solute carrier family 25 member 4 or ANT1; OPA1: OPA1 mitochondrial dynamin like GTPase; NNT: nicotinamide nucleotide transhydrogenase; NNMT: nicotinamide N-methyltransferase; NMRK1: nicotinamide riboside kinase 1; NT5E: 5′-nucleotidase ecto; NAMPT: Nicotinamide Phosphoribosyltransferase; NMNAT1, NMNAT3: Nicotinamide Mononucleotide Adenylyl Transferase 1, 3.

Differentially expressed genes involved in mitochondrial biogenesis and organization and in the maintenance of the NAD+/NADH ratio are illustrated in Figure 7. The mitochondrial biogenesis is dependent on the transcription and the post-transcriptional activation of the proliferator-activated receptor γ coactivator-1 α (PGC1α). PGC1α synthesis and activation are upregulated by the mTORC1, the AMPK, the calcium/calmodulin-dependent kinase, as well as by the estrogen related receptors (ERRα, ERRβ and ERRγ) pathways [54,55]. 

Of note, mTOR directly regulates the transcription of ERRα-target genes involved in energy metabolism [56]. PGC1α, ERRα, ERRβ and ERRγ were all found to be upregulated in the trained subjects with respect to sedentary ones, together with other mitochondrial genes, namely the mitochondrial transcription factor A, most subunits of the mitochondrial complexes, most mitochondrial ribosomal proteins, the adenine nucleotide translocator ANT1, and OPA1. Ubaida-Mohien et al. [26] reported the decline of skeletal muscle mitochondrial proteins with age and their maintenance as a function of exercise level [25]. Protein by protein, there is an almost perfect correspondence between the ageing-related changes described by Ubaida-Mohien et al. [26] and their reversal at the mRNA level in the aged athletes, including isocitrate dehydrogenase IDH2 and IDH3A; moreover, in trained subjects, the transcription of NAD(P) transhydrogenase (NNT)—another NADP+ reducing enzyme, which contributes to the maintenance of proton gradients across the mitochondrial membranes is enhanced.

As a result of mitochondrial dysfunction and age, the NAD+/NADH ratio declines [57]; of particular concern is the maintenance of NAD+ mitochondrial pool, crucial for energy metabolism and cellular viability [58]. Our data suggest that life-long high-level exercise appear to offset the age-related decline of NAD+. In fact, by comparing our results with the reported variation in abundance of NAD-metabolism enzymes which occurs with aging [26], we observed that most age-associated variations (specifically, the increase of NMMT, NMRK1 and NT5E) are reversed in the gene expression of trained subjects. In TRA VL, we also observed an increase in the gene expression of two key enzymes of NAD biosynthesis (nicotinamide phosphoribosyltransferase, and the major isoforms of nicotinamide mononucleotide adenylyl transferase).

All together, these data support the notion that high-level exercise training carried out for many years was able to preserve muscle cell energy level in aged athletes. Several studies (reviewed in refs. [14,59]) reported the beneficial effects of both endurance and resistance training in preserving mitochondrial mass, function and dynamics in skeletal muscles of aging individuals; however, most trials involved a relatively short-term training. A transcriptional study by Melov et al. [22] reported the improvement of both mitochondrial impairment and muscle weakness following a six-month resistance exercise training, thus showing that both goals can be achieved by long-time training.

#### Calcium Involvement in Energy-Related Processes 

**Figure 8:**
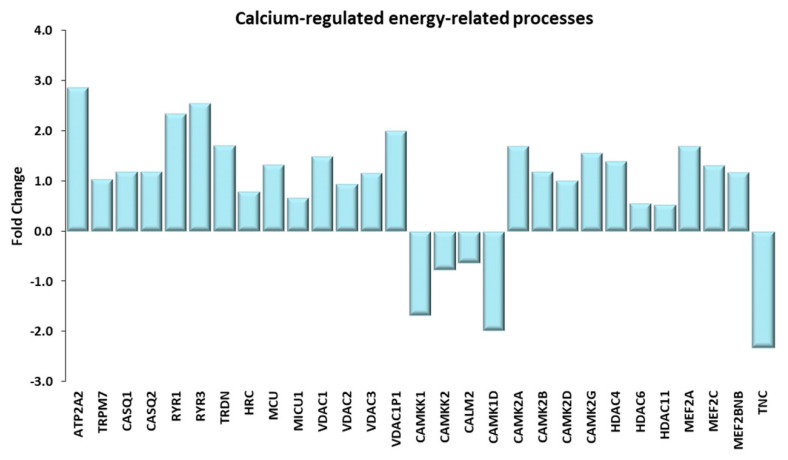
Calcium-regulated energy-related processes. Genes discussed in the text are shown here as fold change in the comparison of TRA and SED subjects. ATP2A2: ATPase sarcoplasmic/endoplasmic reticulum Ca^2+^ transporting 2 or SERCA2; TRPM7: transient receptor potential cation channel subfamily M member 7; CASQ1, CASQ2: calsequestrin 1, 2; RYR1, RYR3: ryanodine receptor 1, 3; TRDN: triadin; HCR: histidine rich calcium binding protein; MCU: mitochondrial calcium uniporter; MICU1: mitochondrial calcium uptake 1; VDAC1, VDAC2, VDAC3, VDAC1P1: voltage dependent anion channel 1, 2, 3, 1 pseudogene 1; CAMKK1, CAMKK2: calcium/calmodulin dependent protein kinase kinase 1, 2; CALM2: calmodulin 2; CAMK1D: calcium/calmodulin dependent protein kinase ID; CAMK2A, CAMK2B, CAMK2D, CAMK2G: calcium/calmodulin dependent protein kinase II α, II β, II δ, II γ; HDAC4, HDAC6, HDAC11 histone deacetylase 4, 6, 11. MEF2A, MEF2C: myocyte enhancer factor 2A, 2C; MEF2BNB: BLOC-1 related complex subunit 8; TNC, tenascin C.

As pointed out above, calcium signaling, required for muscular activity, has a role also in the upregulation of GLUT4 and in the synthesis of PGC1α. Calcium release units (CRU), or triads, specialized membrane structures involved in excitation-contraction coupling (EEC), are physically associated to mitochondria [60] and favor the entry of Ca^2+^ into the mitochondrial matrix, in order to stimulate the respiratory chain and increase production of ATP. In a number of neuromuscular diseases [61] and with muscle ageing [62], massive triad disruption has been described, which compromises both muscle and mitochondrial efficiency. It has been reported that both the triad organization and the mitochondrial number was preserved in the same group of trained elderly we studied [63]. A large array of proteins involved in excitation-contraction coupling was transcriptionally upregulated in TRA VL with respect to SED VL; they include a number of calcium-handling proteins located in the sarcoplasmic reticulum, such as the Ca^2+^ transporter SERCA2A, the Ca^2+^-binding protein calsequestrin, the ryanodine receptor, the membrane-bound tryadin and its partner HRC, which has a role in binding Ca^2+^ inside the sarcoplasmic reticulum. Moreover, the upregulation of mitochondrial calcium uniporter MCU and of mitochondrial calcium uptake 1 MICU1 shows that the entry of Ca^2+^ into the mitochondria was in turn favored by long-term training. Notably, MCU and OPA1 gene upregulation, as well as recovery of mitochondrial mass and ultrastructure, were described to occur also in 70-year-old subjects trained for 9 weeks with either neuromuscular electrical stimulation or leg press [64,65], showing that calcium homeostasis and muscle contraction affect both gene regulation and structural integrity of the EEC and of mitochondria. Differentially expressed genes involved in the above described Ca^2+^-related processes are shown in Figure 8.

#### Thyroid Hormone Signaling Pathway 

**Figure 9:**
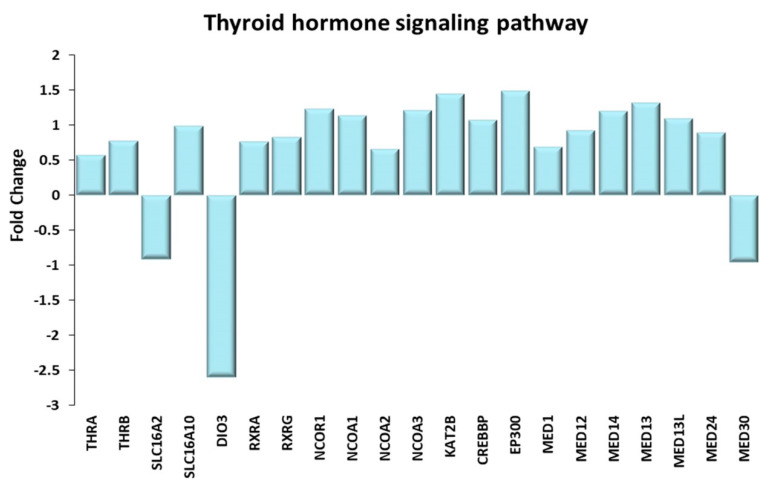
Thyroid hormone signaling pathway. Genes discussed in the text are shown here as fold change in the comparison of TRA and SED subjects. THRA, THRB: thyroid hormone receptor α and β; SLC16A2: solute carrier family 16 member 2 or MCT8; SLC16A10: solute carrier family 16 member 10 or MCT10; DIO3: iodothyronine deiodinase 3; RXRA, RXRG: retinoid X receptor α, γ; NCOR1: nuclear receptor corepressor 1; NCOA1, NCOA2, NCOA3: nuclear receptor coactivator 1, 2, 3; KAT2B: lysine acetyltransferase 2B or PCAF; CREBBP: CREB binding protein or CBP; EP300: E1A binding protein p300 or p300; MED1, MED12, MED13, MED13L, MED14, MED24, MED30: mediator complex subunit 1, 12, 13, 13L, 14, 24, 30 or thyroid hormone receptor (TR)-associated proteins (TRAPs).

Thyroid hormones regulate the energy expenditure of mammalian tissues, hence the skeletal muscle is one of their major targets. Differentially expressed genes of the Thyroid Hormone Signaling Pathway are shown in Figure 9. Thyroxine (T4) and triiodothyronine (T3) regulate metabolism, contractility and myogenesis of the skeletal muscle [66]. The effects of TH on skeletal muscle depend on several factors: (i) the TH serum concentration; (ii) the presence of TH transporters (the monocarboxylate transporters MCT10 and MCT8) on plasma membrane, which control the TH uptake, and (iii) the activity of the two enzymes, the deiodinase type 2 (D2) or 3 (D3) encoded by the DIO2 and DIO3 genes, respectively, which are involved in the interconversion of T4 to T3 and vice versa. D2 converts T4 (the TH inactive form) to T3 (the TH active form), while D3 catalyzes the reverse conversion of T3 to T4. Once activated, T3 binds to the thyroid hormone receptors (THRs), which are encoded by the THRA and THRB genes, leading to gene expression modulation. THRs can exert their function by interacting with the retinoid X receptor (RXR) [67], leading to the formation of a heterodimeric complex. The activation or repression of this complex depends on the presence of T3 ligands and of co-activators, such as NCoA-1, p300/CBP, PCAF, thyroid hormone receptor (TR)-associated proteins (TRAPs), and repressors such as NCoR-1 [67]. It was demonstrated that serum TH concentration decreases with aging [68] and that physical activity induces a transient increase of TH hormones in serum [69,70].

In elderly trained subjects, the gene encoding the MCT10 transporter was upregulated, while that encoding MCT8 was downregulated with respect to sedentary subjects. In their review, Visser et al. [71] reported that “MCT10 enhances intracellular metabolism of T3 to a larger extent than metabolism of T4. Under identical conditions, T3 transport mediated by MCT10 exceeds that by MCT8.” Moreover, TRA subjects downregulated the DIO3 gene, likely leading to a greater availability of the TH active form T3, while upregulating the intracellular T3 receptors, THRA and THRB. While the gene expression pattern of TRA VL suggests that the TH signaling pathway is upregulated by training, it is also clear that it is tightly regulated. In fact, in TRA VL, not only the expression of RXR, but also that of both repressors and co-activators was upregulated.

It is known that T3 stimulates mitochondrial biogenesis and activity and enhances the responsivity to insulin, through the upregulation of GLUT4 expression, thus contributing to the increase in oxidative energy metabolism. This is in keeping with the above-described effects of exercise training in elderly subjects. Bloise FF et al. [68] reported that T3 stimulates slow-to-fast muscle fiber type switch, through the downregulation of slow type I fiber MYH7 expression and upregulation of the fast type II fiber genes. On the contrary, as discussed above, data from this exceptional group of senior athletes point to a training-induced fast-to-slow fiber switch (19). It is possible that these discrepancies are due to the different human model examined.

#### 2.4.2. Proteostasis and Mitophagy 

**Figure 10:**
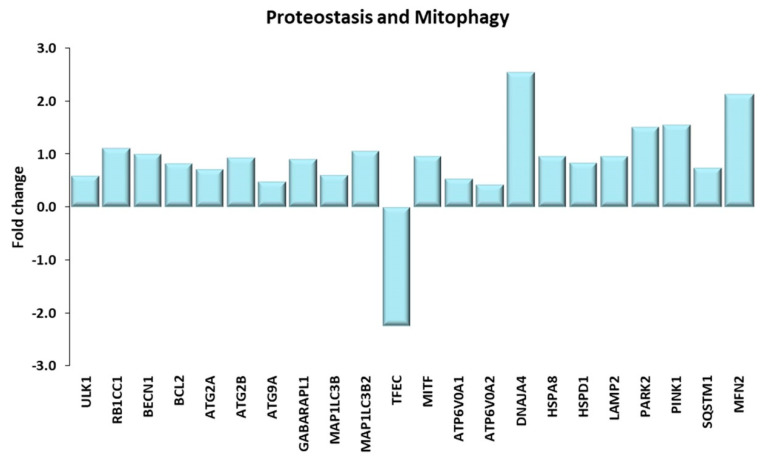
Proteostasis and Mitophagy. Genes discussed in the text are shown here as fold change in the comparison of TRA and SED subjects. ULK1: unc-51 like autophagy activating kinase 1; RB1CC1: RB1 inducible coiled-coil 1 or FIP220; BECN1: beclin1; BCL2: BCL2 apoptosis regulator; ATG2A, ATG2B, ATG9A: autophagy related 2A, 2B, 9A. GABARAPL1: GABA(A) receptor-associated protein like 1; MAP1LC3B, MAP1LC3B2: microtubule associated protein 1 light chain 3 β, β2; TFEC: transcription factor EC; MITF: melanocyte inducing transcription factor; ATP6V0A1, ATP6V0A2: ATPase H+ transporting V0 subunit A1, A2; DNAJA4: DnaJ heat shock protein family (Hsp40) member A4; HSPA8: heat shock protein family A (Hsp70) member 8; HSPD1: heat shock protein family D (Hsp60) member 1; LAMP2: lysosomal associated membrane protein 2; PARK2: parkin RBR E3 ubiquitin protein ligase; PINK1: PTEN induced kinase 1; SQSTM1: sequestosome 1 or p62; MFN2: mitofusin 2.

Differentially expressed genes involved in proteostasis and mitophagy are shown in Figure 10. Autophagy is a process that promotes the degradation of misfolded proteins and damaged organelles [72], playing a crucial role in the maintaining of skeletal muscle homeostasis and integrity, by increasing the regenerative potential of satellite cells [5,73,74]. Macroautophagy and chaperone-mediated autophagy (CMA) are the most characterized autophagy pathways [75]. A complex network of signals, involving the AMPK signaling pathway and mTOR, controls macroautophagy. On one hand, AMPK induces macroautophagy, on the other hand, autophagy is negatively regulated by the mTORC1 complex. Therefore, a tight regulation of these pathways balances their catabolic and anabolic effects on skeletal muscle. Both AMPK and mTORC1 act by phosphorylating the pro-autophagic kinase ULK1 in distinct residues; moreover, mTORC1 blocks the autophagosome formation by an inhibitory phosphorylation of ATG13, a positive regulator of ULK1 [72]. Macroautophagy requires the de novo formation of double membrane structures termed autophagosomes, which sequester damaged proteins and then fuse with lysosomes. Macroautophagy decreases with age [75], however exercise training appears to offset such decline, since we observed an increase in gene transcription of a number of key components of the phagophore (ULK1, FIP200, Beclin1, Bcl2, ATG2A/B, ATG9A, GABARAPL1, MAP1LC3B and MAP1LC3B2) in TRA VL as compared with SED VL.

Lysosomes are necessary to complete the autophagic process. Lysosome biogenesis is controlled through the phosphorylation and inhibition of the MiT/TFE family of transcription factors EB (TFEB), TFE3, TFEC and of the microphthalmia-associated transcription factor (MiTF) [76]. These transcription factors control the expression of lysosomal and autophagy genes; in particular, in Drosophila and in melanocytes, it was demonstrated that MiTF directly controls the transcription of all 15 v-ATPase enzymes [77]. In TRA VL, we found a decrease in the expression of TFEC and—marginally—of TFE3 genes, that may explain the downregulation of most genes of the Lysosome KEGG pathway (see Table S3). On the other hand, MiTF gene expression increased, as well as the expression of two subunits of the v-ATPase pump which are controlled by MiTF. The v-ATPase enzyme pumps protons inside the lysosome, leading to its acidification and to the activation of lysosomal proteases. At variance with literature data, showing that physical exercise fosters the biogenesis of lysosomes [78], our data do not suggest such upregulation, but rather an enhancement of their function and activity by the increase in the expression of pump subunits.

The lysosome functionality is important not only to complete the macroautophagy process but also for CMA pathway. In CMA, the single soluble damaged proteins are recognized by the cytosolic heat shock cognate protein 70 KDa, then the complex substrate-Hsc70 is targeted to the lysosome, where it binds to the lysosome-associated membrane protein LAMP-2A, which allows the internalization of damaged substrates into the lysosome [75]. Ubaida-Mohien et al., [26] demonstrated that the protein expression of different chaperons, including DnaJ homolog subfamily A, HspA8 and Hsp60 protein 1, involved in CMA, are downregulated with age, a condition which may severely impact on the maintenance of proteomic integrity [79]; by comparing the gene expression of these chaperon in sedentary and trained elderly, we found a training-related increase. Moreover LAMP-2, which is required for the autophagosome binding to the lysosomal membrane, was genetically upregulated in TRA muscles with respect to SED ones.

Autophagy processes are initiated by the targeting of proteins or organelles by ubiquitination. The expression of most genes included in the Ubiquitin-mediated proteolysis pathway was upregulated in TRA VL muscle of elderly subjects (see Table S3). In particular, the mRNA abundance of the gene encoding the cytosolic E3 ubiquitin ligase Parkin was increased. Parkin, together with the PTEN-induced kinase 1 (PINK1), plays a crucial role in mitophagy, a particular form of autophagy, which removes damaged mitochondria, thus improving the mitochondrial proteostasis. It is known that mitophagy is impaired with aging and that physical activity prevents aging-related skeletal muscle damage by stimulating autophagy [80,81] as well as mitophagy [82]. Damaged mitochondria recruit PINK1 to the outer mitochondrial membrane (OMM), which, in turn, phosphorylates and activates the Parkin E3 ligase enzyme. Parkin-operated ubiquitylation of several OMM proteins allows their recognition by the adapter protein SQSTM1/p62. P62 interacts with the microtubule-associated protein 1A/1B-light chain 3 (LC3) encoded by MAP1LC3 gene subfamily or with the GABA receptor-associated protein (GABARAP), which are embedded in the double membrane of the phagophore; this step leads to the formation of the autophagosome, which will subsequently fuse with the lysosome [81]. In our model, several proteins involved in this process were upregulated in TRA VL, namely Parkin, PINK1, SQSTM1/p62. Finally, also Mitofusin 2, which stimulates Parkin translocation to the mitochondria, was transcriptionally upregulated [83]. The PINK-Parkin system is one of the two molecular mechanisms which induce mitophagy [81]. All together, these results suggest that autophagy and, in particular mitophagy, are stimulated in elderly subjects by chronic prolonged exercise.

#### 2.4.3. Cytoskeleton 

**Figure 11:**
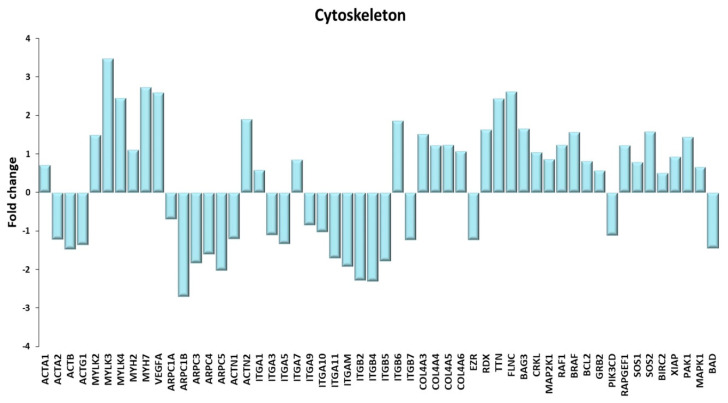
Cytoskeleton. A Genes discussed in the text are shown here as fold change in the comparison of TRA and SED subjects. ACTA1: actin α 1, skeletal muscle; ACTA2: actin α 2, smooth muscle; ACTB: actin β; ACTG1: actin γ 1; MYLK2, MYLK3 myosin light chain kinase 2, 3; MYLK4: myosin light chain kinase family member 4; MYH2, MYH7: myosin heavy chain 2, 7; VEGFA: vascular endothelial growth factor A; ARPC1A, ARPC1B, ARPC3, ARPC4, ARPC5: actin related protein 2/3 complex subunit 1A, 1B, 3, 4, 5; ACTN1, ACTN2: actinin α 1, 2; ITGA1, ITGA3, ITGA5, ITGA7, ITGA9, ITGA10, ITGA11, ITGAM, ITGB2, ITGB4, ITGB5, ITGB6, ITGB7: integrin subunit α1, α3, α5, α7, α9, α10, α11, α M, β2, β4, β5, β6, β7; COL4A3, COL4A4, COL4A5, COL4A6: collagen type IV α3, α4, α5, α6 chain; EZR: ezrin; RDX: radixin; TTN: titin; FLNC: Filamin C; BAG3: BAG co-chaperone 3; CRKL: CRK like proto-oncogene, adaptor protein; MAP2K1: mitogen-activated protein kinase kinase 1; RAF1: Raf-1 proto-oncogene, serine/threonine kinase; BRAF1: B-Raf proto-oncogene, serine/threonine kinase; BCL2: BCL2 apoptosis regulator; GRB2: growth factor receptor bound protein 2; PIK3CD: phosphatidylinositol-4,5-bisphosphate 3-kinase catalytic subunit δ; RAPGEF1: Rap guanine nucleotide exchange factor 1; SOS1, SOS2: SOS Ras/Rac guanine nucleotide exchange factor 1, 2; BIRC2: baculoviral IAP repeat containing 2; XIAP: X-linked inhibitor of apoptosis; PAK1: p21 (RAC1) activated kinase 1; MAPK1: mitogen-activated protein kinase 1; BAD: BCL2 associated agonist of cell death.

The cytoskeleton of the skeletal muscle fiber is organized in four topographically distinct and functional compartments [84]: (i) the contractile apparatus; (ii) the intra-sarcomeric cytoskeleton, providing anchorage and supporting the displacement of the contractile myofilaments; (iii) the peri-sarcomeric and inter-myofibrillar network, assuring the coordination between adjacent sarcomers; (iv) the sub-sarcolemmal cytoskeleton, linking the peripheral myofibrils to the cell membrane and providing anchorage through indirect binding to the extracellular matrix. In the sub-sarcolemmal region, focal adhesion complexes, specifically named costamers in striated muscle, host a number of relevant kinases, the most prominent being Focal Adhesion Kinase, Proline-rich tyrosine kinase, and Integrin-Linked Kinase [85]. The mechanical tasks carried out by the cytoskeleton are associated to a sophisticated system of sensors, relaying information about cytoskeleton loading and muscle tension to the nuclei, to change gene expression. Signals are generated either from one end to the other of the muscle fiber or within the focal adhesion complexes [86]. In association with myosin, actin constitutes the contractile apparatus; moreover, actin coordinates a large host of intermediate filaments and other proteins involved in the maintenance of cell structure, in signal transduction, in cell-to-ECM adhesion and in particle and vesicle transport. Differentially expressed genes are shown in Figure 11.

The muscle-specific Actin A gene was upregulated in TRA VL, while other actin genes, typically expressed in different cell types, were downregulated. The expression of all myosin light chain kinases and of the more relevant myosin genes that characterizes slow-twitch muscles (MYH7 and MYH2) was increased with training, consistent with the fast-to-slow switch already described to have occurred in these subjects [19]. Together with the increase in slow-twitch myosin, we observed increased mitochondrial biogenesis, increased VEGFA gene expression (VEGFA being the major angiogenetic factor), and increased gene expression of SERCA2, the Ca^2+^ ATPase pump isoform specific for slow twitch (as well as for cardiac, and smooth muscle), all results that indicate an increase in muscle oxidative metabolism independently on the training mode (RT vs. ET). Notably, a widespread conception requires that aging involves a fast-to-slow fiber type switch. However, Purves-Smith et al. [87] point to the weakness of evidences supporting such notion and suggest that the occurrence of denervation and other issues may lead to fiber misclassification. Indeed, our data strongly point to a training-induced increase of oxidative muscle fibers in VL of aged subjects, which does not contradict the fact that resistance-trained subjects have larger fast-twitch fibers than endurance-trained ones.

The “regulation of the actin cytoskeleton” KEGG pathway shows gene expression changes in a large number of actin-interacting proteins. In general, exercise (i) downregulated some proteins involved in cell shape changes, such as ARP2/3; (ii) increased the expression of “muscle-specific” isoforms of proteins at the expenses of more nonspecific ones (e.g., ACTN2, the muscle-specific isoform of actinin, was upregulated, while the aspecific isoform ACTN1 was downregulated); (iii) altered the expression pattern of as many as 13 integrins, proteins that in some way characterize cell identity and mediate cell-to-cell and cell-to-ECM adhesion, as well as signals generated at the costamers [85]. In this regard, it is worth mentioning also the increased expression of four isoforms of Collagen A4, a major component of basal membranes, and gene expression changes involving two of the ERM proteins (ezrin and radixin), that act as cross-linkers between the plasma membrane and the actin-based cytoskeleton. Wackerhage et al. [86] mention three proteins associated with the Z band, namely titin, Filamin C and Bag3, which likely mediate muscle-loading signals to the mTOR pathway. They were all upregulated in TRA VL. The focal adhesion KEGG pathway underlines changes in the costamer-related composition and signaling, pinpointing in particular the exercise-induced upregulation of cell survival signals and downregulation of pro-apoptotic genes as BAD.

#### 2.4.4. Inflammation 

**Figure 12:**
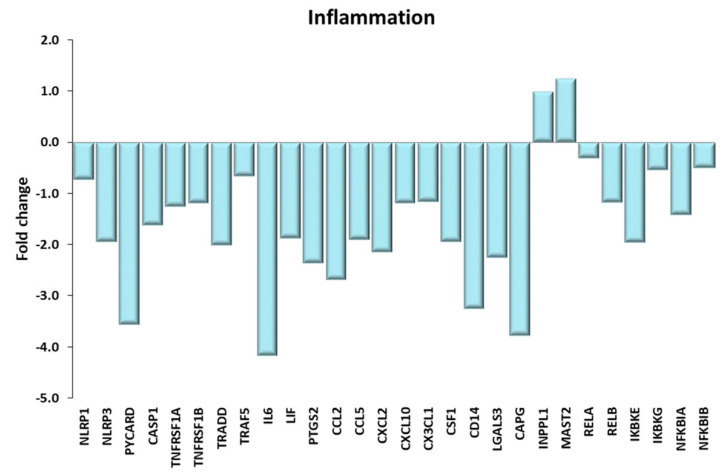
Inflammation. Genes discussed in the text are shown here as fold change in the comparison of TRA and SED subjects. NLRP1, NLRP3: NLR family pyrin domain containing 1, 3; PYCARD: PYD and CARD domain containing or ASC; CASP1: caspase1; TNFRSF1A, TNFRSF1B: TNF receptor superfamily member 1A, 1B; TRADD: TNFRSF1A associated via death domain. TRAF5: TNF receptor associated factor 5; IL6: interleukin 6; LIF: LIF interleukin 6 family cytokine; PTGS2: prostaglandin-endoperoxide synthase 2; CCL2, CCL5: C-C motif chemokine ligand 2, 5; CXCL2, CXCL10: C-X-C motif chemokine ligand 2, 10; CX3CL1: C-X3-C motif chemokine ligand 1; CSF1: colony stimulating factor 1; CD14: CD14 molecule; LGALS3: galectin 3; CAPG: capping actin protein, gelsolin like; INPPL1: inositol polyphosphate phosphatase like 1; MAST2: microtubule associated serine/threonine kinase 2; RELA, RELB: RELA, RELB proto-oncogene, NF-kB subunit; IKBKE, IKBKG: inhibitor of nuclear factor κ B kinase subunit ε, γ; NFKBIA, NFKBIB: NFKB inhibitor α, β.

Several evidences suggest that regular physical activity exerts anti-inflammatory effects in both old and young people [88,89]. On the contrary, it was demonstrated that acute bouts of exercise induce a significant increase of pro-inflammatory molecules [90]. In our study, KEGG analysis identified among the highest scoring pathways the “NOD-like receptor signaling pathway” and the “TNF signaling pathway”, and many pathological pathways, which are related to inflammation. Genes discussed in the present paragraph are shown in Figure 12. The “NOD-like receptor signaling pathway” consists of genes involved in the innate immunity and in the inflammasome formation. Inflammasomes are multimeric protein complexes formed by an inflammatory sensor molecule belonging the NOD-like receptor (NLR) family proteins (NLRP1, NLRPL3 and NLRC4), the adaptor protein ASC encoded by the Pycard gene and the pro-Caspase 1. In response to pathogen-associated molecular patterns (PAMPs) and damage-associated molecular patterns (DAMPs), the NRLs assemble together to form a platform—the inflammasome, leading to the activation of the pro-caspase 1; in turn, caspase-1 directly cleaves pro-IL-1β and pro-IL-18, leading to complete maturation of both pro-inflammatory cytokines [91,92]. In VL of lifelong trained elderly subjects, we found that the gene expression of NLRP1, NRPL3, ASC and Caspase 1 was downregulated with respect to SED. The decreased expression of the inflammasome genes following exercise has been already described to occur in both young and old men [93,94] who underwent a training period that did not exceed three months.

As for the “TNF signaling pathway”, we found that the two TNF-alpha receptor genes (TNFRSF1A and TNFRSF1B), as well as the genes encoding the adaptor proteins TRADD and TRAF5, which play a crucial role in the TNF-α signaling transduction, were downregulated with training. In addition, several genes controlled by the TNF-α signaling pathway were downregulated in TRA VL compared to SED, in particular two pro-inflammatory cytokines (IL-6 and LIF) and PTGS2, which is involved in the synthesis of inflammatory mediators. Moreover, the genes involved in the recruitment (CCL2, CCL5, CXCL2, CXCL10 and CX3CL1) and activation (CFS1) of leucocytes were downregulated in TRA VL of elderly subjects.

Noteworthy, we found that the gene expression of most age-associated pro- and anti-inflammatory proteins described by Ubaida-Mohien et al. [26] had the opposite trend in TRA subjects. Specifically, some proteins (Cd14, LGALS3, CAPG), which are related to a pro-inflammatory state and are upregulated in elderly subjects, were decreased in VL TRA, while the gene expression of two NF-κB attenuators, INPPL1 and MAST2, which are down-regulated with aging [26], resulted upregulated in VL TRA. In addition, the expression of the NF-kB subunits REL-A and REL-B was decreased in TRA VL compared to SED, despite the counterintuitive downregulation of their inhibitors. Overall, our data suggest an attenuation of the NF-kB pathways and of the age-associated inflammatory status as a consequence of long-life exercise training.

#### 2.4.5. Cellular Senescence 

Cellular senescence differs from organismic senescence in that it involves single cells, which are mainly characterized by cell growth arrest, reduced autophagy and synthesis of a characteristic pro-inflammatory secretome and of proteases [95]. Cellular senescence may be triggered by telomere dysfunction or by the concurrent presence of an unresolved DNA damage response (DDR) associated with growth factor signaling, which ultimately turns out to be a conflict between simultaneous p53 and mTOR signaling. In aging individuals, the presence of connective tissue cells characterized by senescence-associated secretory phenotype (SASP) may affect neighboring muscle tissues by paracrine secretion, promoting a dangerous pro-inflammatory environment and decreasing the regenerating capacity of muscle satellite cells [96]. It has been shown that athletes, in particular older individuals, tend to have longer telomere lengths than non-athletes [97].

**Figure 13:**
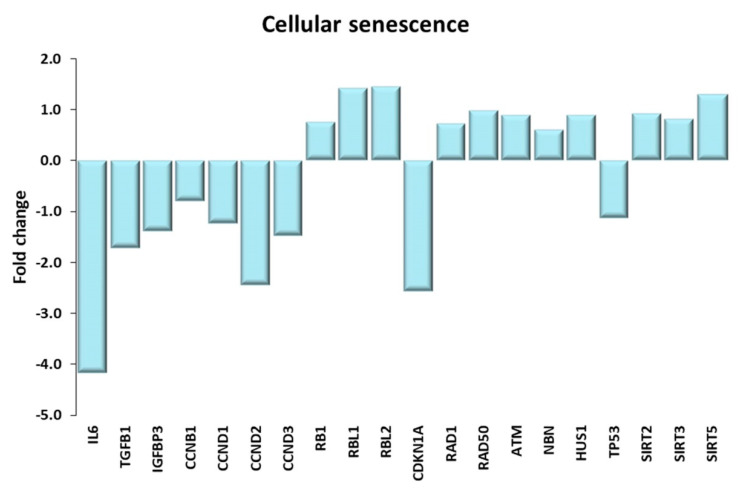
Cellular senescence. Genes discussed in the text are shown here as fold change in the comparison of TRA and SED subjects. IL6: interleukin 6, TGFB1: transforming growth factor beta 1; IGFBP3 insulin like growth factor binding protein 3; CCNB1, CCND1, CCND2, CCND3: cyclin B1, D1, D2, D3; RB1: RB transcriptional corepressor 1; RBL1, RBL2: RB transcriptional corepressor like 1, 2. CDKN1A: cyclin dependent kinase inhibitor 1A; RAD1: RAD1 checkpoint DNA exonuclease; RAD50: RAD50 double strand break repair protein; ATM: ATM serine/threonine kinase; NBN: nibrin; HSU1: HUS1 checkpoint clamp component; TP53: tumor protein p53; SIRT2, SIRT3, SIRT5: sirtuin 2, 3, 5.

As shown in Figure 13, in our transcriptomics study, all the components of the senescent secretome listed in the KEGG pathway were downregulated in trained muscles, in particular the pathognomonic cytokine IL-6 [98]. Moreover, as discussed above, components of the autophagy process were upregulated by exercise training. The pivotal role of autophagy in preventing muscle cell senescence has been recently underlined [73,99].

There are many cues suggesting that trained muscle cells were more subject to cell cycle arrest processes than those of sedentary subjects; in fact, gene expression of four cyclins (CCNB1, CCND1–3), was downregulated, and that of three out of four cell-cycle inhibitors (RB1, RBL1, RBL2) was upregulated (the exception being CDKN1A) in TRA VL. However, the specific cell cycle inhibitor characterizing DDR, eventually leading to cellular senescence, is p16Ink4a (CDKN2A), which was not expressed. As for DDR, some components, such as RAD1, RAD50, ATM, NBN, HUS1 were found to be upregulated in TRA VL. Actually, prolonged exercise may generate reactive oxygen species, a possible cause of telomere shortening and DNA damage, and hence of DDR. DDR, in turn, leads to the transcriptional upregulation of p53, a very important player in the cellular senescence pathway. Full p53 activation then requires crucial post-translational modifications of the protein [100]. Yet, p53 was transcriptionally downregulated by training. On the other side, mTOR was transcriptionally upregulated in trained muscle cells, however, as noted above, it was only partially activated by training-related insulin-like signals, since these were counteracted by opposite inactivating signals generated by the AMPK pathway.

Overall, the general picture emerging from these data suggests that the final effect of life-long high-level exercise training on muscle senescence in the elderly leans toward the counteracting of the senescent phenotype. Literature data on the effect of exercise training in contrasting muscle cell senescence are relatively few, and most address specific mechanisms, such as autophagy [99] or AMPK activation [101]. A study on young human subjects reported the presence of senescent endothelial cells within the skeletal muscle and their clearance following resistance training [102]. Sirtuins, a family of NAD-dependent deacetylases, have been implicated in favoring longevity in mammals and may play a role also in reducing cellular senescence (reviewed in ref. [103]). We observed a transcriptional increase in most SIRT isoforms in TRA VL, likely as a result of the hypothesized NAD^+^ increase that is brought about by life-long high-level exercise training (discussed above).

#### 2.4.6. Final Remarks

The senior athletes studied here have made high-level training part of their lifestyle. This feature makes the present study unique, since most published studies involving the effect of exercise on skeletal muscles were carried out on subjects who were exposed to a relatively short-term training. Few authors [25,26,104] studied long-term active subjects; however, the exercise intensity level did not reach with that of our senior athletes. This may explain some unforeseen results we report here, such as the fact that the training mode (ET vs. RT) was not relevant for the pathways leading to the prevention of sarcopenia. However, we cannot rule out that this is because we omitted to standardize the timing of biopsies, which might have resulted into inhomogeneous expression of circadian-regulated genes of ET TRA subjects. The comparison of our data with the transcriptomics ones reported by Ubaida-Mohien et al. [25,26] was unexpectedly fruitful, for in most instances we found a perfect inverse relationship between the aging-related proteins reported therein and the differential gene expression observed in aged individuals as a result of long-term high-level training. In fact, while we expected that the final outcome of exercise training in old age was the contrast to sarcopenia, we did not anticipate that in chronic high-level exercise the very same pathways and processes leading to loss of muscle mass and energy would have been involved in reversing the aging process. In fact, it was shared opinion that “… human muscle age-related molecular processes appear distinct from the processes regulated by those of physical activity” [28], an opinion that we challenge in the case of these exceptional senior athletes.

## 3. Materials and Methods

### 3.1. Subjects

Control sedentary subjects were chosen among patients undergoing elective knee surgery at the Second Orthopaedic and Traumatologic Clinic, IRCCS Istituto Ortopedico Rizzoli, Bologna, Italy. Inclusion criteria were: male; between 65 and 79 years of age; sedentary lifestyle (physical activity limited to carrying out a minimum level of daily routine due to knee pain). Exclusion criteria were: being affected by metabolic, degenerative, inflammatory, autoimmune, infectious or tumoral diseases; having undergone surgery in the last three months; taking anabolic or doping drugs and dietary supplements. Their anthropometric features are summarised in Table 1. Biopsies were obtained concurrently with a surgical procedure carried out in proximity of the biopsy itself and consisted of about 20–25 mg of muscle tissue taken from VL, approximately 20 cm above the knee. Patients were fasting; surgery was carried out in the morning, but no standardization of biopsy timing was pursued. The bioptic fragment was immediately dipped in RNAlater™ solution (Invitrogen, Milan, Italy). The purposes of the study and the details relating to the biopsy were explained to the candidate participants, which subsequently signed an informed consent form. The nstudy was approved by the Ethical Committee of the IRCCS Istituto Ortopedico Rizzoli (Prot. gen. n.ro 0002659).

Senior sportsmen (*n* = 9) were exceptional amateur athletes who had trained at high level for at least 30 years and continued routinely to practice, usually more than three times a week, with a total workload of 10.1 ± 5.84 h/week (mean ± SD). With the exception of the exercise practice, inclusion and exclusion criteria were the same as those applied to sedentary elderly. Their anthropometric features, the type and amount of exercise practiced and muscle performance are summarized in Table 2 and Table 3. As detailed in Table 2, four of them were predominantly resistance trained, whereas five were predominantly endurance trained. Muscle biopsies were obtained through a small skin incision (6 mm) from VL and immediately frozen in isopentane cooled in liquid nitrogen, then stored at −80 °C until use. Subjects were instructed to avoid exercising in the 24 h preceding the biopsy; no standardization of biopsy timing was pursued. All participants were informed on the purposes of the study and on the details of the biopsy and signed an informed consent form. The study was approved by the Ethical Committee of the City of Vienna (EK-08-102-0608). Methods for evaluating the anthropometric features and the muscle performance are detailed in refs [18,19].

### 3.2. Light Microscopy and Quantitative Histological Analyses

Serial cryosections (8 µm) from frozen muscle biopsies were mounted on Polysine™ glass slides, air-dried and stained either with hematoxylin and eosin (H&E), or with conventional techniques for activity of myofibrillar ATPases to evaluate muscle fiber type. Histology and subsequent morphometric analyses were performed on stained cryosections as described [105]. To overcome the distortion of obliquely cut or kinked muscle fibers, a condition commonly observed in human muscle biopsies, we measured the lesser myofiber diameter as previously described [106]. The mean myofiber diameter from a total of 200 fibers in each biopsy was calculated, and a comparison between ET and RT trained subjects using Student t-test by GraphPad PRISM 7.0a (GraphPad Inc., La Jolla, CA, USA) was performed. Differences among groups were considered statistically significant when the *p* value was less than 0.05; the exact *p* values are reported in the legend of Table 4.

### 3.3. RNA Extraction and Quality Control

After collection, all muscle samples were processed within two weeks. Samples stored in RNAlater™ solution were rinsed in buffered saline and frozen in liquid nitrogen; frozen samples were reduced to powder using a sterilized ceramic mortar and pestle. Total RNA was extracted with TRIZOL^®^ (Invitrogen SRL, Milan, Italy) according to the manufacturer’s instructions [107].

### 3.4. NGS

Total RNA concentration was measured by spectrophotometer (Ultrospec 2000, Pharmacia Biotech, Uppsala, Sweden). The RNA purity was assessed by measuring the OD260/280 ratio which ranged between 1.85 and 2.03 (mean 1.98). RNA integrity was assessed by evaluating the 28S and 18S band sharpness after denaturing electrophoresis [108,109]. Furthermore, densitometric analysis of 28S and 18S bands was performed using the Quantity One software (Bio-Rad Laboratories, Hercules, CA, USA). The mean of the intensity ratio of the 28S and 18S bands was 1.8. No difference in RNA quality was found between samples collected in RNAlater™ or frozen immediately after the biopsy.

Whole-transcriptome RNA libraries were prepared in accordance with Illumina’s TruSeq RNA Sample Prep v2 protocol (Illumina, San Diego, CA, USA). Poly(A)-RNA molecules from 500 ng of total RNA were purified using oligo-dT magnetic beads. Following purification, the mRNA was fragmented and randomly primed for reverse transcription followed by second-strand synthesis to create double-stranded cDNA fragments. These cDNA fragments went through a terminal-end repair process and ligation using paired-end sequencing adapters. The products were then amplified to enrich for fragments carrying adapters ligated on both ends and to add sequences complementary to the oligonucleotides on the flow cell, thus creating the final cDNA library.

cDNA library size was checked and sized with Agilent DNA 1000 chips on the Bioanalyzer 2100 (Agilent Technologies, Taoyuan County, Taiwan), then libraries were quantified using PicoGreen assay (Life Technologies, Carlsbad, CA, USA). 12 pM paired-end libraries were amplified and ligated to the flowcell by bridge PCR, and sequenced at 2 × 80 bp read length for RNA using Illumina Sequencing by synthesis (SBS) technology.

### 3.5. NGS Data Analysis

After quality check and trimming, paired-end reads were mapped on human reference genome with the Tophat/Bowtie alignment pipeline. PCR and optical duplicates were removed with samtools. The gene expression was quantified using the python function Htseq-count (assuming the Ensembl GRCh37 v72 as gene annotation) and normalized as count per million (cpm) with the R-bioconductor package edgeR. The analysis of differential gene expression was performed adopting the limma R-bioconductor package (lmFit and eBayes functions) on the set of expressed genes (in each comparison the genes with at least 2 cpm in at least 2 samples).The results were reported in Excel format; columns report Gene name (Ensembl gene ID and Official gene symbol), LogFC, nominal *p*-value, adjusted *p*-value (AKA q-value, computed by Benjamini-Hochberg correction) and cpm of each sample.

### 3.6. Unsupervised Analysis

Mixture distribution analysis clustered the studied subjects according to logical trees based on the similarity of gene expression, thus leading to mixture coefficient maps. It was performed in unsupervised mode, according to the non-negative matrix factorization (NMF) method, which, starting from cpm, clusters the samples and the genes in a number of pre-selected groups, which are suggested by a preliminary step on the basis of a coefficient and with the help of a heatmap (R package NMF—https://cran.r-project.org/web/packages/NMF/index.html).

The method allows the identification of the set of genes, which mostly characterize each group. Accordingly, cluster analysis was first applied on the assumption that data will be clustered in two groups, i.e., TRA and SED. Then, it was applied assuming the presence of three groups, i.e., SED, RT and ET.

### 3.7. Pathway Analysis

Pathway analyses were performed on the list of differentially expressed genes, which were subsequently screened only based on their *p* value (*p* ≤ 0.01). This allowed a better identification of the involved pathways. In fact, within a single pathway, genes may vary to a considerably different extent, yet they all contribute to the involvement of a given pathway in a complex biological process.

The selected genes were evaluated by the Kyoto Encyclopedia of Genes and Genomes (KEGG, Release 91.0, 1 July 2019) using Enrichr, an integrative web-based software (https://bmcbioinformatics.biomedcentral.com/ articles/10.1186/1471-2105-14-128) to elucidate their relevant functions. Fisher’s exact test *p*-values were calculated to identify which functional gene groups were significantly enriched.

Within the first 25 highest scoring pathways, we restricted our analysis to 15 pathways based on their relevance to the biological model studied here. In fact, the pathways we decided to not analyze concern human diseases and/or were inappropriate for skeletal muscle. In our opinion, they resulted from the KEGG analysis because they involve inflammation and/or cell survival mechanisms; however, we take into consideration these issues in the subsections “Inflammation” and “Cellular senescence”. The first 25 highest scoring pathways are shown in Table S2 with all details provided by the KEGG analysis.

## Figures and Tables

**Table 1:** Anthropometric characteristics of healthy sedentary subjects.

Code	Age (years)	Height (cm)	Body Mass (kg)	Body Mass Index (BMI)
L167	70	181	81	24.7
L178	76	173	77	25.7
L179	72	160	83	32.4
L180	75	178	89	28.1
L181	71	170	78	27.0

BMI is defined as the body mass divided by the square of the body height (kg/m^2^).

**Table 2:** Anthropometric and training characteristics of trained subjects.

Code	Age (years)	Height (cm)	Body Mass (kg)	BMI	Resistance Training (%)	Endurance Training (%)	Mean Duration of Sessions (hrs)	Sessions/Week (no.)	Duration/Week (hrs)
L182	68.5	169.5	81.6	28.4	100	0	1.5	3	4.5
L183	65.0	184.0	99.0	29.2	89	11	3.0	5	9.0
L184	70.6	174.0	78.5	25.8	100	0	2.3	6	6.9
L185	69.3	184.0	87.0	25.7	65	35	2.0	3	6.0
L186	68.2	176.0	73.6	23.8	13	87	2.0	4	8.0
L187	65.5	174.2	73.4	24.2	0	100	3.0	8	14.0
L188	66.0	174.0	76.0	25.1	14	86	1.5	4	6.0
L189	66.8	180.0	93.0	28.7	14	86	5.5	13	24.5
L190	79.7	170.0	67.0	23.2	0	100	2.0	6	12.0
Mean ± SD	68.84 ± 4.5	176.19 ± 5.4	81.01 ± 10.3	26.01 ± 2.2			2.53 ± 1.2	5.78 ± 3.1	10.1 ± 6.2

Shaded boxes correspond to subjects with a prevalent resistance training. Body Mass Index (BMI) is defined as the body mass divided by the square of the body height (kg/m^2^).

**Table 3:** Muscle performance of trained subjects.

Code	Knee Extension Torque Left Leg (N∙m)	Knee Extension Torque Right Leg (N∙m)	Knee Extension Torque Left Leg (N∙m/kg)	Knee Extension Torque Right Leg (N∙m/kg)
L182	147.0	176.0	1.80	2.16
L183	215.0	215.0	2.17	2.17
L184 *				
L185	142.0	196.0	1.63	1.07
L186	164.0	202.0	2.23	2.74
L187	167.0	175.0	2.28	2.38
L188	164.0	192.0	2.16	2.53
L189	191.0	215.0	2.05	2.31
L190	157.0	175.0	2.34	2.61
Mean ± SD	168.38 ± 23.9	193.25 ± 16.9	2.08 ± 0.2	2.25 ± 0.5

* Subject L184 did not show up to functional measurement test. Shaded boxes correspond to subjects with a prevalent resistance training. N∙m = Newton by meter.

**Table 4:** Mean myofiber diameter calculated on skeletal muscle biopsies from RT and ET trained subjects.

Fiber Diameter	RT	ET	*p* Value
**Overall** (mean ± SD)	70.30 ± 8.7	58.6 ± 6.6	**0.0129**
**Slow type** (mean ± SD)	66.14 ± 7.1	63.9 ± 6.4	0.5180
**%**	68	67	
**Fast type** (mean ± SD)	74.62 ± 9.9	56.27 ± 8.1	**0.0019**
**%**	32	34	

**Table 5:** Genes characterizing the two groups of trained subjects.

Gene Name	logFC edgeR	logCPM edgeR	*p* Value edgeR	FDR edgeR	logFC Limma	AveExpr Limma	t Limma	*p*.Value Limma	adj.*p*.Val Limma	L187	L188	L189	L182	L183	L184	L185
NR4A3	5.58938	8.05454	0.00000	0.00006	5.84709	6.64827	12.82643	0.00000	0.00086	610.02	880.91	1891.64	29.65	8.81	19.26	19.24
ANKRD1	5.44798	9.39036	0.00000	0.00001	6.18242	7.78585	13.94543	0.00000	0.00049	4183.80	1110.87	3514.04	38.07	22.20	39.67	45.05
ATF3	5.30250	8.04398	0.00000	0.00220	6.16321	6.54035	19.07142	0.00000	0.00023	1513.03	685.26	1144.91	19.77	9.18	18.19	14.85
OTUD1	4.66495	9.20151	0.00000	0.00598	5.61890	7.95328	14.97490	0.00000	0.00037	3795.33	1300.29	2408.51	33.82	41.22	78.98	42.52
FOS	4.32165	8.69905	0.00006	0.03627	5.53600	5.84895	11.70230	0.00000	0.00166	820.07	383.46	435.27	6.70	11.47	6.30	30.46
XIRP1	3.73963	11.72048	0.00000	0.00089	4.47180	11.11266	15.02730	0.00000	0.00037	18,249.37	7680.84	15,550.17	566.93	457.31	546.19	822.20
HBEGF	3.49074	6.92300	0.00002	0.01783	4.50824	6.13966	10.76029	0.00000	0.00215	769.71	237.69	401.67	18.46	15.76	12.42	31.39
MYC	3.40054	5.54740	0.00000	0.00089	4.18256	4.66004	15.03225	0.00000	0.00037	182.95	93.34	132.65	7.68	5.70	9.14	6.92
IER5	3.32338	7.68888	0.00001	0.01181	3.97225	7.12766	14.22916	0.00000	0.00048	883.14	393.64	869.56	36.60	41.37	53.42	41.43
CYR61	3.32101	7.30011	0.00008	0.04099	4.47312	6.08245	8.95268	0.00001	0.00448	891.86	202.52	339.93	20.50	10.58	25.20	18.81
NR4A1	3.25759	7.89653	0.00002	0.01783	3.65943	7.50826	15.68667	0.00000	0.00037	586.58	787.75	994.26	46.65	51.14	82.17	71.38
BTG2	3.13166	8.92563	0.00003	0.02371	3.61149	8.46996	10.98758	0.00000	0.00210	1825.79	755.04	2331.93	124.91	111.09	141.27	108.59
ENAH	3.04548	8.97210	0.00003	0.01977	3.74105	8.59328	8.12615	0.00002	0.00661	2844.32	707.23	2407.53	111.83	85.63	139.59	192.28
CSRNP1	2.79483	5.52248	0.00008	0.04099	3.62103	4.88653	10.18447	0.00000	0.00263	194.28	71.28	134.73	9.39	8.44	12.33	10.38
NFIL3	2.71683	5.92826	0.00000	0.00089	3.17555	5.65796	10.36495	0.00000	0.00241	183.04	116.54	258.44	12.50	19.69	23.16	25.65
ARRDC4	2.27807	5.64728	0.00004	0.02555	3.04853	5.50522	10.40753	0.00000	0.00241	227.91	105.32	145.64	22.71	20.94	11.89	19.41
RAB15	2.00799	4.71411	0.00010	0.04384	2.51144	4.76790	9.78657	0.00000	0.00305	82.59	48.65	97.42	12.91	10.21	13.93	14.85
B3GNT5	1.97126	6.45269	0.00009	0.04256	2.51777	6.53882	9.65582	0.00001	0.00305	337.24	150.67	311.67	49.01	45.00	38.25	43.54
GADD45A	1.92487	5.14147	0.00002	0.01783	2.41089	4.46737	8.53077	0.00001	0.00568	85.12	39.41	56.07	13.15	12.14	9.67	8.52
CD300LG	1.32879	4.94671	0.00004	0.02708	1.31297	4.97485	6.37281	0.00014	0.01987	52.10	44.97	61.64	19.85	14.80	26.71	25.82

ET Subjects correctly classified by mixture distribution analysis (bright blue background) vs. RT Subjects (green background).

**Table 6:** Genes responsible of the wrong assignment of the ET subjects to their group (shaded genes are also in Table 5).

Gene Name	logFC Limma	AveExpr Limma	t Limma	*p*.Value Limma	adj.*p*.Val Limma	L187	L188	L189	L186	L190
HBEGF	4.16469	7.05932	8.42677	0.00006	0.09556	769.71	237.69	401.67	18.94	29.60
ARRDC4	2.74324	6.15656	8.31871	0.00006	0.09556	227.91	105.32	145.64	21.99	23.45
ANKRD1	4.48285	9.52091	8.74260	0.00005	0.09556	4183.80	1110.87	3514.04	123.13	104.22
IER5	3.78093	7.89060	8.76015	0.00004	0.09556	883.14	393.64	869.56	34.57	70.45
OTUD1	5.55686	8.94707	10.97613	0.00001	0.06737	3795.33	1300.29	2408.51	31.39	76.81
XIRP1	3.56566	12.23974	8.18312	0.00007	0.09556	18,249.37	7680.84	15,550.17	740.04	1607.97
DDN	1.58921	6.14486	9.41066	0.00003	0.09493	123.19	111.54	95.67	38.79	33.97
MAOA	−3.20244	5.34823	−10.15969	0.00002	0.07575	27.36	11.22	14.84	152.31	156.95
Xxbac-B476C20.17	2.157934	5.3604339	11.274756	8.26 × 10^−6^	0.06737	83.55	66.85	74.84	18.81	14.49

ET Subjects correctly classified by mixture distribution analysis (bright blue background) vs. ET Subjects misclassified by mixture distribution analysis (light blue background).

**Table 7:** Relevant highest scoring KEGG pathways.

Term	Overlap	*p*-Value	Adjusted *p*-Value	Number of Genes Up-Regulated in TRA VL vs. SED VL
Insulin signaling pathway	80/137	3.10 × 10^−7^	9.56 × 10^−5^	54/80
Lysosome	72/123	1.05 × 10^−6^	1.61 × 10^−4^	17/72
Focal adhesion	105/199	4.39 × 10^−6^	3.38 × 10^−4^	48/105
Ribosome	84/153	5.21 × 10^−6^	3.21 × 10^−4^	10/84
Adipocytokine signaling pathway	44/69	6.09 × 10^−6^	2.34 × 10^−4^	25/44
Regulation of actin cytoskeleton	110/214	1.26 × 10^−5^	4.30 × 10^−4^	45/110
AMPK signaling pathway	66/120	4.74 × 10^−5^	1.22 × 10^−3^	41/66
Thyroid hormone signaling pathway	64/116	5.40 × 10^−5^	1.28 × 10^−3^	44/64
Citrate cycle (TCA cycle)	22/30	5.90 × 10^−5^	1.30 × 10^−3^	20/22
Insulin resistance	60/108	6.98 × 10^−5^	1.34 × 10^−3^	35/60
Ubiquitin mediated proteolysis	73/137	7.85 × 10^−5^	1.42 × 10^−3^	55/73
TNF signaling pathway	60/110	1.40 × 10^−4^	2.16 × 10^−3^	22/60
NOD-like receptor signaling pathway	89/178	2.80 × 10^−4^	3.93 × 10^−3^	33/89
Cellular senescence	81/160	3.12 × 10^−4^	4.17 × 10^−3^	43/81

## Supplementary Materials

Supplementary Materials can be found at https://www.mdpi.com/1422-0067/21/11/3988/s1. Table S1: Complete NGS results; A: TRA vs. SED; B: ET vs. RT. Table S2: 25 highest-scoring KEGG pathways identified. Table S3: Differentially expressed genes in the relevant KEGG pathways.

## Abbreviations

SED	sedentary
TRA	trained
VL	vastus lateralis muscle
ET	endurance training
RT	resistance training
KEGG	Kyoto Encyclopedia of Genes and Genomes
NGS	Next Generation Sequencing
TCA	tricarboxylic acid or citric acid, or Krebs cycle
mTOR	mechanistic Target Of Rapamicyn
CMA	chaperone-mediated autophagy
OMM	Outer Mitochondrial Membrane
DDR	DNA Damage Response.
